# Depleting Autoreactive B‑Cells Using Targeted Photodynamic Therapy

**DOI:** 10.1021/acsptsci.5c00332

**Published:** 2025-09-20

**Authors:** Kevin R. Venrooij, Theodoros Ioannis Papdimitriou, Daphne N. Dorst, Kimberly M. Bonger

**Affiliations:** † Institute of Molecules and Materials, Synthetic Organic Chemistry, 6034 Radboud University, Heyendaalseweg 135, Nijmegen 6525 AJ, The Netherlands; ‡ Department of Rheumatology, Radboudumc, Geert Grooteplein Zuid 8, Nijmegen 6525GA, The Netherlands; § Leiden University, Leiden Institute for Chemistry, Chemical Biology & Immunology, Einsteinweg 55, CC Leiden 2333, The Netherlands

**Keywords:** autoimmune disease, antigen-selective B-cell targeting, multivalent antigens, photosensitizer, targeted photodynamic therapy

## Abstract

In many autoimmune pathologies, including Rheumatoid Arthritis (RA), only a small percentage of the total B cell population is autoreactive and sustain disease. Yet, current immunotherapy treatments often eliminate the entire B-cell population, leading to immune deficiency. We developed an approach to selectively eliminate autoreactive B cells with targeted photodynamic therapy (tPDT). We designed a construct containing a dimeric peptidic antigen (diCCP4) that selectively binds a patient-derived autoreactive B cell receptor (BCR) and additionally included the photosensitizer IRDye700DX. We tested the construct on a modified Ramos B-cell line (Ramos 3F3), expressing this specific autoreactive BCR sequence. After brief exposure to 689 nm light, the photosensitizer selectively eliminates the modified Ramos cells, while the construct is not cytotoxic to cells lacking the autoreactive BCR. In a 3D coculture of the Ramos autoreactive B cell line with peripheral blood mononuclear cells (PBMCs) we observed only a minimal response of the untargeted cells. These results highlight the potential of tPDT against autoreactive B cells in autoimmune disease.

## Graphical abstract

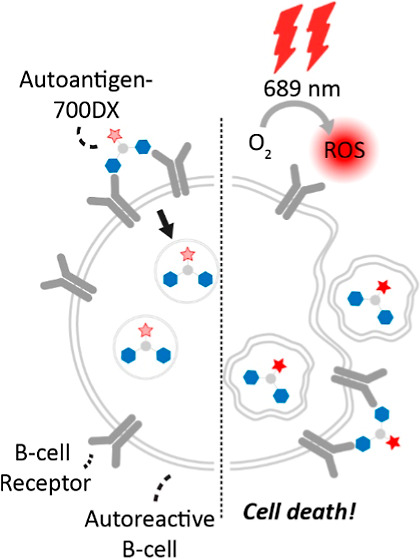

Rheumatoid Arthritis (RA) is an autoimmune disease that affects about 1% of the population in industrialized countries.^1^ The symptoms of RA include joint inflammation, stiffness and bone erosion. Autoreactive B cells play a central role in disease progression by secreting autoantibodies.^2^ Analysis of peripheral blood mononuclear cells (PBMCs) from RA donors revealed that these self-reactive B cells comprise approximately 0.01% of the total B cell population.^3^ About 70–80% of RA patients harbor anticitrullinated protein antibodies (ACPAs),^4^ which are produced by antibody-secreting plasma cells differentiated from B cells. RA is commonly treated with disease-modifying antirheumatic drugs (DMARDs).^5,6^ As small-molecule chemical DMARDs (cDMARDs) lose their efficacy over time, patients often receive two or three drugs simultaneously.^7–9^ Patients who fail to respond to cDMARDs can be treated with biological DMARDs, for example, rituximab, an anti-CD20 antibody. Rituximab targets and eliminates CD20 positive B cells, leading to alleviation of symptoms.^10^ Long-term studies have shown that rituximab treatment causes low serum immunoglobulin levels in about 4/100 patient-years.^11^ In such cases, the depletion of the entire B cell repertoire can lead to immune deficiency, which increases the risk for severe opportunistic infections.^5,6^

A promising avenue for RA treatment is the specific elimination of autoreactive B cells.^12–14^ We propose a targeting strategy using a synthetic autoantigen, specifically targeting the autoreactive B cell receptor (BCR), coupled to an effector molecule with cytotoxic properties. Synthetic cyclic citrullinated peptides (CCPs) have been used as autoantigen in diagnostics to detect and target autoreactive B cells in the context of RA.^15–18^ Previously, we have shown that a synthetic dimer of CCP4 (diCCP4) selectively targets a transfected Ramos B cell line expressing a patient-derived BCR sequence recognizing citrullinated proteins (Ramos-3F3).^17^ diCCP4 rapidly binds and internalizes into the Ramos-3F3 cells in a nanomolar concentration range, while monomeric CCP4 peptide binds more than 3 orders of magnitude lower and did not internalize at the concentration tested.^17^ We further demonstrated that, likely due to the short interpeptide distance, diCCP4 was hardly sequestered by free floating 3F3 antibodies compared multivalent CCP4 peptides that were spaced further apart. Because of these properties, we envisioned to combine the excellent targeting properties of diCCP4 with the locoregional advantage of targeted photodynamic therapy (tPDT) (Figure 1).

**Figure 1:**
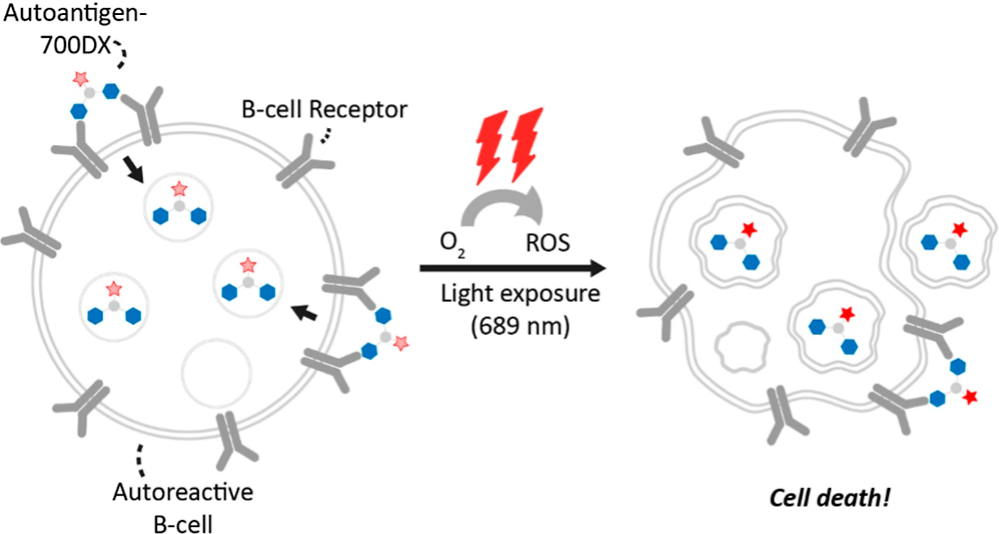
Conceptual overview: autoreactive B cells bind synthetic dimeric autoantigen-IRDye700DX construct through the B cell receptor and internalizes into the cell. Upon exposure to 689 nm light, the IRDye700DX (red start) produces local reactive oxygen species (ROS) causing toxicity and selective elimination of autoreactive B cells.

tPDT is a two-step therapy that uses a light sensitive photosensitizer (PS) molecule to produce cytotoxic reactive oxygen species (ROS) upon exposure of light. Several in vivo studies have applied (nontargeted) PDT in the context of RA. Compounds such as protoporhyrin IX,^19^ [Na]­[ATX-S10][Bibr ref20] and Talaporfin^21^ preferentially accumulated in the synovium of inflamed joints. After irradiation, this resulted in reduced inflammation and less damage to the cartilage, which highlights the potential of light therapy in joints.^22^ However, despite preferential accumulation of the PS in metabolically active cells, synovial tissue often suffers from necrosis.^22^ Dorst et al. showed that conjugation of the PS IRDye700DX to a full-size antibody allowed for targeted PDT (tPDT) of pathogenic fibroblasts in preclinical models of arthritis, pancreatic cancer and systemic sclerosis.^23–26^ tPDT enables both site- and marker- specific targeting of pathogenic cell-types, preventing off target cytotoxicity.

In this study, we conjugated the synthetic autoantigen diCCP4 to the IRDye700DX photosensitizer to form diCCP4–700DX.^27^ We evaluated the cytotoxicity and specificity of diCCP4–700DX on the Ramos-3F3 cell model. Ramos-TT cells, containing a BCR that recognizes an unrelated tetanus-toxoid (TT) antigen, served as a control cell line.^3,17^ Additionally, we seeded the Ramos-3F3 or -TT cells in a 3D matrix using collagen type-I to increase the complexity of our model and mimic tissue structure more accurately. Finally, we investigated the potential host immune response to cell death following autoantigen-tPDT (bystander effect) on Ramos-3F3 cells by coculturing them with peripheral blood mononuclear cells (PBMCs) from healthy donors.

## Methods

### Synthesis of diCCP4–700DX

diCCP4-NH_2_ was synthesized as previously described by van Weijsten et al.^17^ diCCP4-NH_2_ (54 μL of a 520 μM stock solution in 50 mM phosphate buffer, pH 7.3; 1.1 equiv) was diluted with 20 μL of DMSO. Subsequently, IRDye700DX-NHS (Licor, 10 μL of a 2.5 mM solution in anhydrous DMSO; 1.0 equiv) was slowly added and mixed with a pipet. Finally, an additional 36 μL phosphate buffer (50 mM, pH 7.3) was added and the mixture was left in an Eppendorf shaker overnight. After 16 h, 17 μL DMSO was added to improve the solubility. The suspension was then diluted with 64 μL phosphate buffer (50 mM, pH 7.3) and subsequently centrifuged to remove insoluble particles (RT, 1 min, 3000*g*). The resulting clear teal supernatant contained diCCP4–700DX (50 μM, 23.1% DMSO in 50 mM phosphate buffer, 200 μL) and the yield of 52% was determined with UV–vis at 689 nm with the extinction coefficient of IRDye-700DX (ε = 165,000 ^28^). The solution was then aliquoted, snap frozen with liquid nitrogen and stored at −80 °C. LCMS (Rt) 7.36 min. LCMS (ESI+) *m*/*z*: calcd for C_247_H_369_N_78_O_66_S_8_Si_3_, [M+3H]^3+^ 1941.17; found, 1942.24. C_247_H_370_N_78_O_66_S_8_Si_3_, [M+4H]^4+^ 1456.13; found, 1457.24. C_247_H_371_N_78_O_66_S_8_Si_3_, [M+5H]^5+^ 1165.10; found, 1165.96.

### General Cell Culture

The production of Ramos-3F3 and TT cells were previously described.^29^ The cells were cultured in RPMI 1640 with Hepes and GlutaMax (FisherScientific, 11544526), which was supplemented with heat shocked 10% FCS OneShot (FisherScientific, 155955309) and 1% penicillin/streptomycin in standard culture conditions (37 °C, 5% CO_2_). Cells were cultured with a density between 0.25 and 2.5 × 10^6^ cells/mL.

### DiCCP4–700DX tPDT Cytotoxicity Experiments in 2D Monoculture

100.000 Ramos-3F3 cells were seeded in a 96 well flat bottom plate (Corning, ref3596) and incubated with diCCP4–700DX for indicated incubation time and concentration at 37 °C, 5% CO_2_. The cells were then centrifuged (5 min, 300 rcf) and washed twice with complete medium and exposed to 689 nm light at a fluency rate of 290 mW/cm^2^ using an LED light source (LEDfactory, Leeuwarden, The Netherlands). After 16 h incubation at 37 °C, 5% CO_2_ the viability was measured with the CellTiter-Glo Luminescent Cell Viability Assay (CTG, Promega, G756A) in black 96 well plates (Corning, ref3915) on a Tecan Spark M10 plate reader.

In the diCCP4–700DX dose titration experiments the cells were treated with 0–100 nM diCCP4–700DX or 0.05% DMSO vehicle, and incubated for 90 min and treated with 50 J/cm^2^ 689 nm light. In experiments regarding the incubation time of diCCP4–700DX the time was varied between 0, 1, 5, 15, 30, 60, and 90 min and cells received 10 nM diCCP4–700DX and 50 J/cm^2^. Light dose experiments used 10 nM diCCP4–700DX and 90 min incubation time, but varied the light dose between 0 and 150 J/cm^2^.

### Coculture of PBMCs and Ramos-3F3 Cells

Peripheral blood mononuclear cells (PBMCs) were isolated from buffy-coats obtained from healthy donors that consented to donate blood for medical research (Sanquin bloodbank, project number NVT 0397–02) using a Ficoll–Paque gradient as previously described.^30^ Collagen type I plugs with Ramos-3F3 cells and PBMCs were prepared with 20 μL MEM, 10 μL BIC, 150 μL collagen (PureCol collagen type I, Advanced BioMatrix, #5005) and 90 μL cell suspension per well while on ice. Each plug contained 2 × 10^5^ PBMCs and 2 × 10^4^ Ramos-3F3 cells. This suspension was then seeded into a 48 well plate (Cellstar, 677180) and left in the incubator for 1 h at 37 C and 5% CO_2_ to solidify. After solidification, 750 μL culture medium, with or without 30 nM diCCP4–700DX, was added and the plugs were incubated overnight in standard culture conditions. The next day the plugs were washed with PBS, exposed to 689 nm light (50 J/cm^2^, 290 mW/cm^2^) and left to incubate again overnight. The next morning the plugs were detached and digested as described above. They were then stained for flow cytometry using the antibody panel described in Supporting Information Table 1. Briefly, after digestion the cells were washed with cold PBS (4 °C, 300*g*, 5 min) twice. Fixable viability dye was added and incubated on ice for 30 min in the dark. The cells were subsequently washed once with cold PBS and once with cold FACS buffer (FB, PBS with 1% bovine serum albumin (BSA, Sigma)). The antibody mixture (Supporting Information. Table 1) was added and incubated for 20 min at RT in the dark. Cells were subsequently washed three times with FB (4 °C, 300*g*, 5 min). The cells were then fixed with 1% formalin in PBS for 10 min on ice, in the dark. Finally the cells were washed with FB, resuspended in FB and transferred to FACS tubes. Samples were then measured on the CytoFLEX LX 21 flow cytometer (Beckman Coulter) and analyzed using Kaluza analysis software (Beckman Coulter, version 2.1). Results are depicted as the percentage positive population.

### Statistics

Results are shown as mean (s.d.). The statistical significance was tested using unpaired Student’s *t*-test or two-way ANOVA with Tukey correction for flow cytometry data in GraphPad Prism software (version 5.03; GraphPad Software, San Diego, CA, USA). A *P*-value <0.05 was considered significant.

## Results

### Synthesis of diCCP4–700DX

The synthesis of diCCP4–700DX (**6**) (Figure 2., full structure in Supporting Information Figure 1) started with the generation of CCP4 (**2**), that we used as targeting autoantigen peptide.^17^ In brief, we used Fmoc solid phase peptide synthesis (SPPS) to synthesize the linear precursor form of CCP4 (**1**). Upon completion, we removed the N-terminal Fmoc group and functionalized the N-terminus with chloroacetic anhydride. We cleaved the peptide from the resin under strong acidic conditions and cyclized the peptide via the chloride on the N-terminus and the side chain of cysteine under dilute and mild basic conditions. Next, we reacted the single free amine on the lysine side chain with azidoacetic acid using NHS chemistry, resulting in CCP4-N_3_ (**3**). To create amine-functionalized dimeric CCP4 (diCCP4-NH_2_, **5**), we utilized a triazin core bearing two alkyne groups and a free amine group (**4**) and reacted it with two CCP4-N_3_ molecules via copper-catalyzed click chemistry (CuAAC). At this stage, we can modify the construct to contain any desired functional group via NHS chemistry, since it only contains a single primary amine.

**Figure 2:**
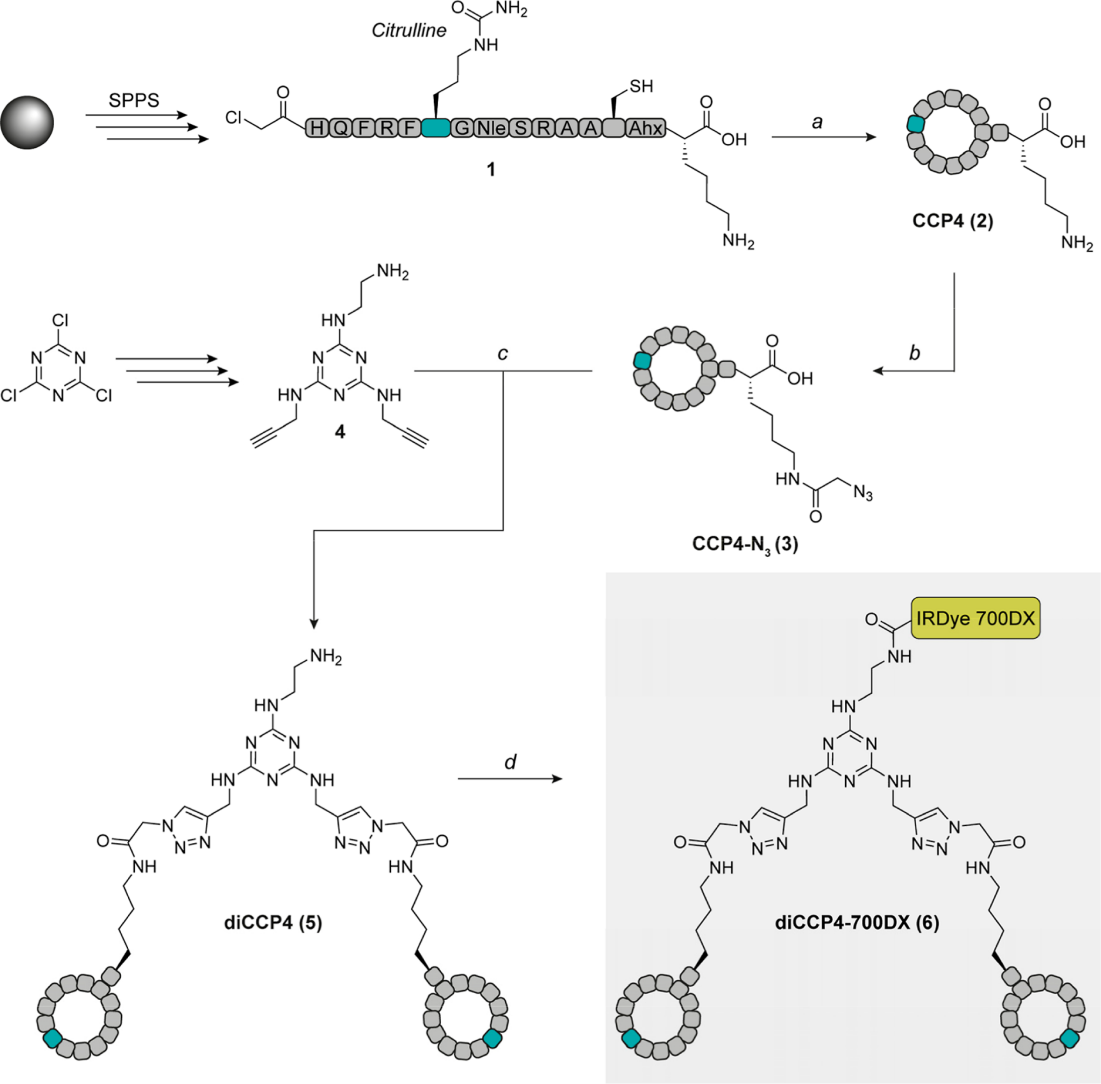
Synthesis scheme of autoantigen diCCP4–700DX (**6**). The linear CCP4 peptide **1** was prepared using Fmoc solid phase peptide synthesis using standard procedures, reacted with chloroacetic anhydride and acidic cleavage from the resin. (a) cyclization of **1** at low concentration (2 mg/mL) in 50 mM NH_4_CO_3_ buffer/ACN (1:1). (b) Azidoacetic acid NHS ester, DIPEA, DMSO. (c) **4**, THPTA, CuSO_4_, sodium ascorbate, Milli-Q. (d) IRDye700DX-NHS, 50 mM phosphate buffer pH 7.3, DMSO.

We opted for IRDye-700DX as payload due to its high potency and proven track record in tPDT. The excitation wavelength of 689 nm suits our application, since this does not induce cytotoxicity and has optimal tissue penetration. We functionalized diCCP4-NH_2_ (1.1 equiv) with IRDye-700DX-NHS in a phosphate buffer with 25% DMSO at room temperature for 16 h. Some precipitate was observed, likely due to the loss of the silyl ether solubilizing arms of the 700DX, which was removed by centrifugation. LC–MS analysis showed that the supernatant contains the desired product, and a 5% impurity of diCCP4-NH_2_ without photosensitizer (Supporting Information Figures 1 and 2).

### DiCCP4–700DX Selectively Eliminates Ramos-3F3 after 689 nm Exposure

Next, we tested our construct on an engineered autoreactive Ramos-3F3 B-cell line, expressing a patient-derived BCR sequence that recognize cyclic citrullinated peptides as previously described.^29^ In this cell line, the genes encoding the endogenous IgM and IgD heavy and light chain as well as the activation-induced cytidine deaminase (AID) protein were deleted to obtain Ramos MDL-AID KO cells. The MDL-AID KO cells were then transduced with an IgG sequence derived from ACPA positive patient, creating Ramos-3F3 B cells.^29^ We used the Ramos MDL-AID KO cells and as well as Ramos cells transduced with a tetanus toxoid IgG sequence (Ramos-TT cells)^3^ as control cells.

Both Ramos-3F3 and Ramos-TT cell lines were incubated with 0–100 nM of diCCP4–700DX for 90 min and subsequently exposed to a moderate amount of 689 nm light (50 J/cm^2^; 3 min). We observed no toxicity to the control Ramos-TT cells, whereas the Ramos-3F3 cells showed dose-dependent cytotoxicity in the nanomolar range (Figure 3A). Moreover, light exposure itself did not induce cytotoxicity, even after 3-fold longer exposure (Supporting Information Figure 3). We observed a similar amount of cytotoxicity using the XTT assay as well as the CTG assay, confirming our results (Supporting Information Figure 4).

**Figure 3:**
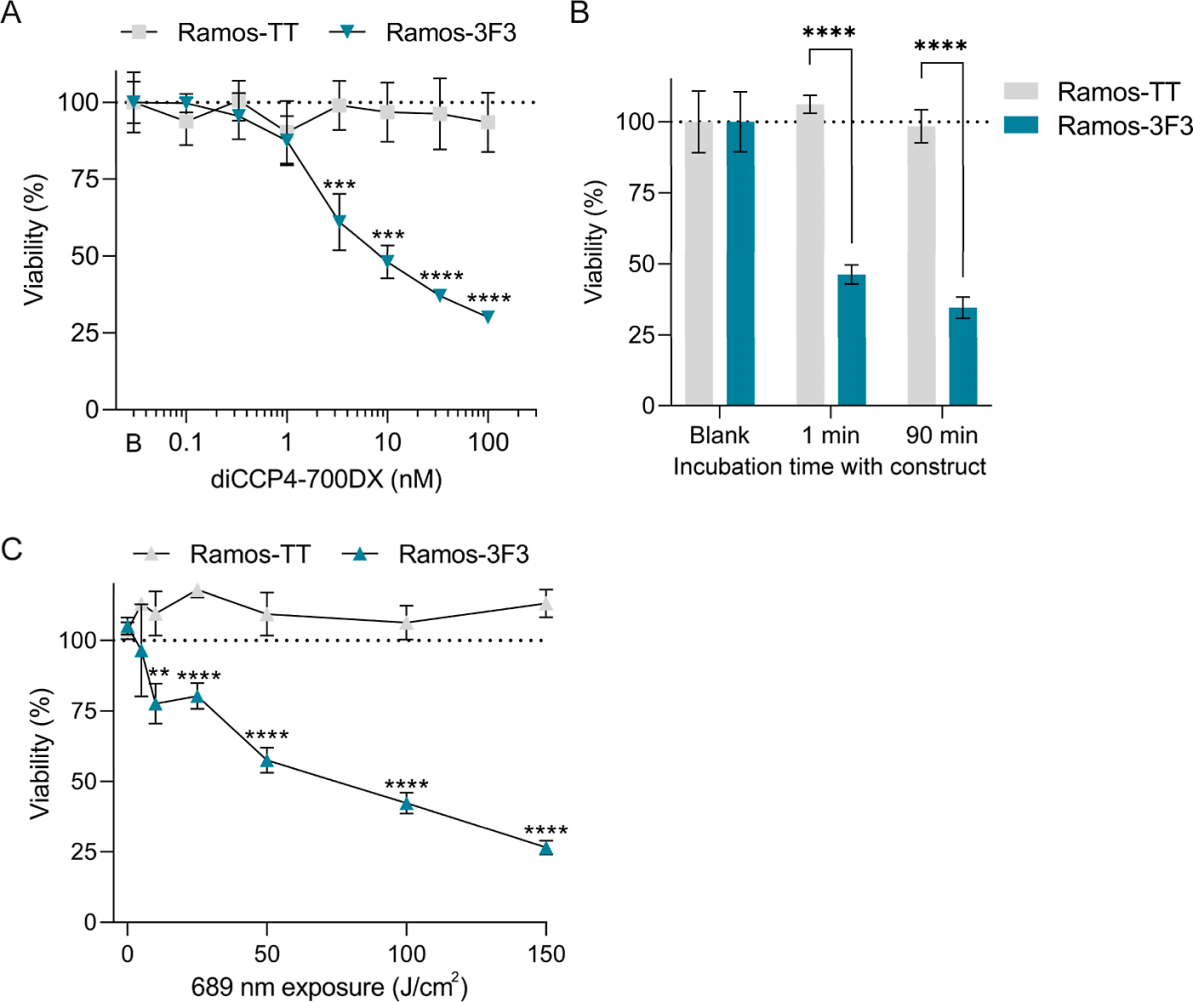
Cytotoxicity of diCCP4–700DX construct on Ramos-3F3 and control Ramos-TT cells. (A) Ramos-3F3 cells are selectively eliminated compared to control Ramos-TT cells with 3 nM or higher concentration. B denotes the vehicle control. (B) Ramos-3F3 cells showed similar cytotoxicity at 10 nM diCCP4–700DX for 1 and 90 min incubation time and subsequent exposure to 689 nm light (50 J/cm^2^), whereas Ramos-TT cells remained viable. Normalized to vehicle blank for each cell line. (C) Cytotoxicity increases by increased exposure to 689 nm light (0–150 J/cm^2^) after treatment with 10 nM diCCP4–700DX for 90 min. (A–C): *n* = 4, experiments are representative examples. **p < 0.01, **p < 0.001, ****p < 0.0001.

In PDT, the extent of local accumulation of the PS and the intensity of light at the specific wavelength determine the overall cytotoxicity. In our previous work we observed nanomolar binding and internalization of diCCP4 within 30 min^17^ Given the promising results observed with diCCP4–700DX after 90 min, we shortened the incubation time. Decreasing the incubation time from 90 to 1 min resulted in a similar amount of cytotoxicity at 10 nM of our construct (Figure 3B). Furthermore, increasing the light intensity further exacerbated cell death in a dose-dependent manner (Figure 3C).

### Elimination of Ramos-3F3 Cells in a Collagen Hydrogel

The extracellular matrix (ECM) is a complex environment and the efficacy of diCCP4–700DX might be affected by its ability to penetrate the ECM and accumulate on autoreactive B-cells or by the penetration depth of the light. To examine this in more detail, we seeded Ramos-3F3 in collagen type I hydrogel to mimic the ECM. We found that diCCP4–700DX induced limited cytotoxicity to Ramos-3F3 cells in the hydrogel compared to similar cell suspension conditions with 90 min incubation. However, increasing the incubation time from 90 min to 16 h restored the cytotoxicity of diCCP4–700DX (Figure 4). Indeed, we confirmed by flow cytometry that diCCP4 conjugated to a sulfo-Cy5 fluorophore internalized slower in Ramos 3F3 cells when growing in a collagen hydrogel compared to cells that were grown in suspension (Supporting Information Figure 5).

**Figure 4:**
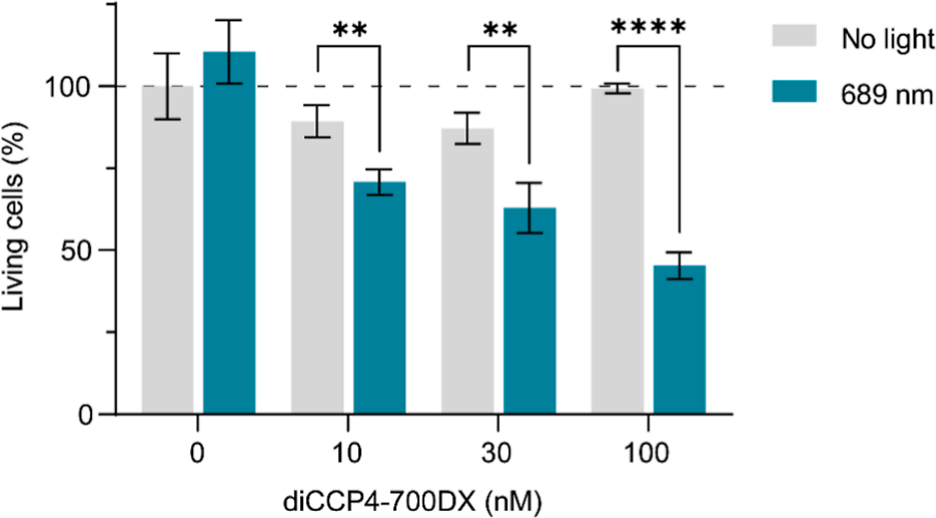
Cytotoxicity in Ramos-3F3 cells in collagen hydrogel after 16 h incubation with diCCP4–700DX and 689 nm light (50 J/cm^2^). **p < 0.01, ****p < 0.0001.

### diCCP4–700DX Selectively Eliminates Ramos-3F3 Cells in the Presence of Healthy Donor PBMC’s

Since the RA synovium contains many different (immune) cells, we next determined the effect of diCCP4–700DX tPDT on neighboring cells. For this, we cocultured Ramos-3F3 cells with healthy donor PBMCs in a collagen type-I matrix and investigated the cytotoxicity and expression markers of diCCP4–700DX with and without illumination on all cells using flow cytometry. The gating strategy is outlined in Supporting Information Figures S6 and S7.

In this setup, diCCP4–700DX combined with 50 J/cm^2^ of 689 nm light (280 mW/cm^2^, 3 min), eliminated 94% of the Ramos-3F3 cell population (Figure 5A). After diCCP4–700DX tPDT treatment, the total cell population almost exclusively consists of donor PBMCs (Figure 5B). Further analysis into the NK cells and T lymphocyte populations showed that treatment with diCCP4–700DX tPDT did not significantly affect the ratiometric distribution of these cell types (Figure 5C,G). For B lymphocytes, although not statistically significant, the diCCP4–700DX tPDT treatment resulted in a slight reduced presence of total B lymphocytes (Figure 5D). NK cell numbers (identified by their expression of CD56) were very low in the collagen plugs. CD56 expression in PBMCs in normal medium was much higher, indicating that digestion of the plugs may interfere with marker expression (Supporting Information Figure 7).

**Figure 5:**
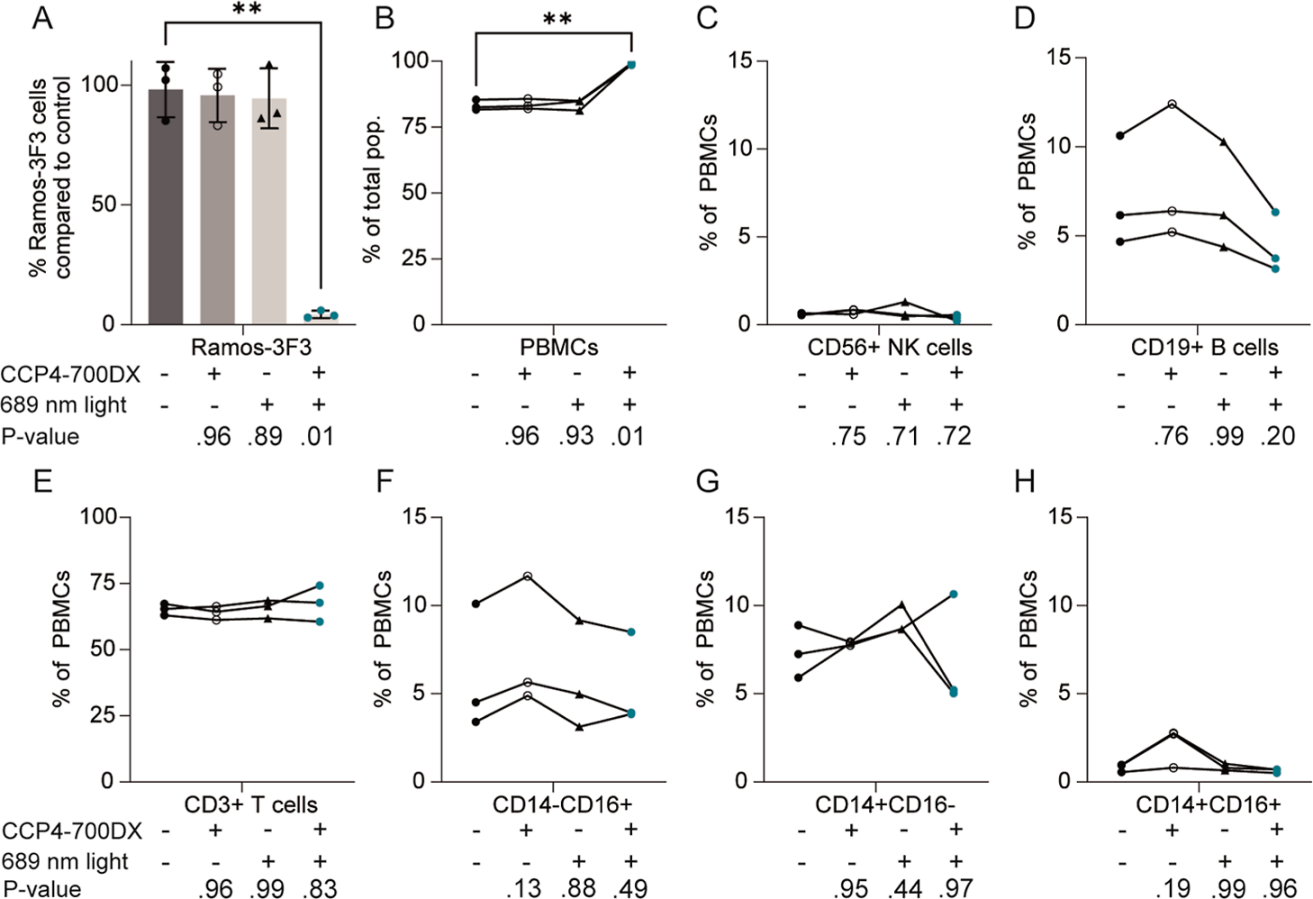
Ramos-3F3 cells cocultured with healthy donor PBMCs in collagen type I hydrogel. Conditions with diCCP4–700DX were incubated with 30 nM construct for 16 h and light conditions received 50 J/cm^2^ of 689 nm light. (A) Ramos-3F3 cells exhibit a lower viability after treatment. (B) The percentage of living PBMC’s is significantly increased due to elimination of Ramos-3F3 cells. (C) Percentage of CD56+ NK cells of the total PBMC population. (D) Percentage of CD19+ B cells of the total PBMC population. (E) Percentage of CD3+ T cells of the total PBMC population. The percentage of monocyte subpopulations CD14-CD16+ (E), CD14+CD16- (F) and CD14+CD16+ (G) out of the total PBMC population. *p ≤ 0.05, **p ≤ 0.01.

Analysis of the B lymphocyte surface markers showed a slight, but nonsignificant, decreased expression of B-cell activation receptor (BAFF-R), a pro-survival receptor in B cells^31^ (Supporting Information Figure 8A). We observed a similar trend in the absence of Ramos-3F3 cells (Supporting Information Figure 9D). The CD19+ B cell population additionally showed a nonsignificant increase in expression of early activation marker CD69 after addition of diCCP4–700DX, regardless of light exposure or presence of Ramos-3F3 cells (Supporting Information Figures 8C and 9E). The nonsignificant reduction of BAFF-R and increase in CD69 suggests that healthy donor B-cells slightly respond to diCCP4–700DX, albeit not in a light-dependent manner.

Next, we determined the extent of T-cell activation by measuring CD69 and CD25 expression, which are early and late T-cell activation markers, respectively.^32–34^ Treatment with diCCP4–700DX nonsignificantly increased CD69 levels and significantly decreased CD25 levels in the CD3+ T cell population, independent of light exposure (Supporting Information Figure 8C,D). We observed no changes in CD69 and CD25 expression in the absence of Ramos-3F3 (Supporting Information Figure 9G,H). This suggests that diCCP4–700DX interacts with Ramos-3F3 cells and influences T cell activation markers in a light-independent manner.

Finally, we examined the effect of diCCP4–700DX tPDT on phagocytic innate immune cell populations. These cell types are predominantly involved in scavenging both exogenous molecules, as well as cell fragments of dead or dying cells. We distinguished the phagocytic cells from the general PBMC population based upon CD14 and CD16 expression. CD16 (FcyRIIIA) is a receptor that recognizes IgG containing immune complexes,^35^ while CD14 is a coreceptor for the detection of pathogens.^36^ CD14^–^CD16^+^, CD14^+^CD16^–^ and CD14^+^CD16^+^ monocyte subpopulations, representing classically-, nonclassically- and intermediately- activated monocytes, respectively (Figure 5F–H).^37–39^ The CD14^low^CD16^+^ monocyte population is known to be increased in the peripheral blood of RA patients.^40^ Monocytes upregulate CD64 in inflammatory conditions.^41^ Interestingly, addition of diCCP4–700DX significantly reduced expression of CD64, an activation marker, of the CD14^–^ CD16^+^ monocyte population, but this was independent of illuminating cells with light (Supporting Information Figure 9E–G). Other monocyte populations did not show significant changes in CD64 expression, nor was it observed in the absence of Ramos 3F3 cells (Supporting Information Figure 9L–N).

Together, these results suggest that healthy donor PBMCs show a limited response to the elimination of Ramos-3F3 cells by diCCP4–700DX combined with illumination with 689 nm light. More specific, PBMCs often responded similarly to either light treatment or diCCP4–700DX treatment alone compared to the combined treatment, indicating that Ramos-3F3 cell death is not the main cause of variation. It should be noted, however, that RA patients often have an activated immune system,^42^ which may respond differently to the cytotoxicity of autoreactive B cell death.

## Discussion

This study provides a proof of principle for the targeting of autoreactive B cells via tPDT. An advantage of tPDT is its local activation, reducing systemic side-effects. However, that also limits the treatment region to superficial or hollow surfaces. A small percentage of autoreactive B cells are present in the circulation and lymph nodes,^3^ which are too widespread or require a high penetration depth for this noninvasive method. To reach these B cells we could expand the scope of the diCCP4 by using effector molecules other than a photosensitizer, such as toxic drug cargo as employed in many antibody-drug conjugate strategies.^43^ Such a strategy would not require an additional light source for activation, allowing the targeting of autoreactive B cells also outside the joint.

Autoimmune diseases such as RA generally contain a wide range of autoantigens, which limits the scope for a single autoantigen as targeting group. Nevertheless, we note that a significant part of the RA B-cell population recognizes citrullinated antigens.^3,44^ Moreover, the high modularity of our system can serve as a solution to this problem, since the autoantigens in our therapeutic construct can be easily adjusted. Next to the targeting moiety, we could also change the photosensitizer. However, tissue penetration is key here and 689 nm light penetrates about 60% deeper into tissue than 550 nm light.^45^ Nevertheless, the estimated 5 mm penetration at 700 nm^45^ suggests that this treatment is only feasible for interphalangeal joints. Treatment of a knee joint will require other methods, like endoscopic delivery of light.^46^

The Ramos-3F3 model overexpresses the autoreactive BCR, which may not accurately reflect the BCR expression levels in patient autoreactive B cells. As a result, diCCP4–700DX might accumulate less on primary autoreactive B cells, leading to reduced cytotoxicity. This could be overcome by increasing the number of PS per diCCP4 molecule. We have opted for a 1:1 ratio of PS and dimeric targeting moiety, whereas often multiple PS are coupled per antibody-700DX construct.^23^ The limited size of the peptidic targeting antigen used here limits the number of PS that can be coupled, since including multiple photosensitizers likely results in challenging physiochemical properties such as solubility.

While Ramos-3F3 cells serve as a robust model for studying autoreactive BCR signaling and antigen specificity, we acknowledge that these cells overexpress the autoreactive BCR derived from patient clones. This overexpression may lead to altered sensitivity to therapeutic agents compared to physiological levels seen in primary B cells. Nonetheless, Ramos-3F3 cells are valuable for mechanistic studies proof-of-concept studies and high-throughput screening, all key objectives of the current manuscript. We acknowledge that for successful transition to the clinical settings it remains critical to validate key observations—such as antigen reactivity, cytokine responses, and therapeutic sensitivity—in primary autoreactive B cells isolated from patients. However, such an approach is highly challenging primarily due to the very low frequency of autoreactive B cells in patients’ blood, where based on our experience we can isolate 500–1000 cells from 50 mL of blood.

To our knowledge, the work described here is the first report of a tPDT construct for the antigen-selective elimination of autoreactive B cells. We observed rapid binding of our construct on Ramos-3F3 cells and subsequently cytotoxicity upon exposure to 689 nm light. Moreover, in a more complex environment with a collagen type I hydrogel and coculturing of Ramos-3F3 cells with PBMCs, we observed that we could eliminate up to 94% of the Ramos-3F3 cells by increasing the incubation time while non autoreactive primary B cells were not significantly affected to the combined therapy.

The cocultured PBMCs displayed a minimal response to targeted photodynamic therapy where the slightly significant changes observed in T cell and monocyte activation are also observed when these cells are exposed to diCCP4–700DX alone. The increase in CD69+ T cells and decrease in CD25+ T cells may result from autoreactive B cell elimination, which affects Tregs that typically express high levels of CD25, as seen in mouse models of autoimmune arthritis where autoreactive B cells enhance Treg numbers.^47^ These subtle off-target effects may result from bystander activation or low-level release of inflammatory mediators following targeted B cell death. In the context of in vivo application, such effects could potentially be mitigated by the spatial restriction of light exposure and rapid clearance of dying cells by phagocytes. Furthermore, transient immune activation might not be detrimental and could even be beneficial in some autoimmune contexts by promoting regulatory mechanisms. Nonetheless, careful optimization of light dosage, construct concentration, and delivery method will be critical to minimizing off-target responses. Future in vivo studies in relevant autoimmune models, particularly RA, will be essential to assess the extent and clinical relevance of these effects under physiological conditions. Our approach demonstrates the potential of specifically targeting and eliminating autoreactive B cells with only a limited effect on neighboring healthy immune cells and underscores the potential of autoantigen-based therapy for other autoimmune diseases.

## Supplementary Material



## Notes

Additional experimental procedures and figures are provided in the Supporting Information (PDF)
