# Supplementary material for: Depleting Autoreactive B‑Cells Using Targeted Photodynamic Therapy
Source: ACS Pharmacol Transl Sci. 2025 Sep 20;8(10):3523–30. doi: 10.1021/acsptsci.5c00332 (PMC12519280; doi:10.1021/acsptsci.5c00332)
Supplement: Supplementary file 1 [file pt5c00332_si_001.pdf]

Supporting Information for

Depleting autoreactive B-cells using targeted photodynamic therapy

Kevin R. Venrooij,[1] Theodoros Ioannis Papdimitriou,[2] Daphne N. Dorst,[1,2] Kimberly M. Bonger.[1,3*]

[1] Radboud University, Institute of Molecules and Materials, Synthetic Organic Chemistry, Heyendaalseweg 135, 6525 AJ, Nijmegen, the Netherlands

[2] Radboudumc, Department of Rheumatology, Geert Grooteplein Zuid 8, 6525 GA Nijmegen, the Netherlands

[3] Leiden University, Leiden Institute for Chemistry, Chemical Biology & Immunology, Einsteinweg 55, 2333 CC Leiden, the Netherlands

* To whom correspondence should be addressed: k.m.bonger@lic.leidenuniv.nl

This document includes:

1. Supplemental experimental procedures
2. Supplemental figures

S-1

1. Supplemental experimental procedures

Comparison cell viability results with XTT and CellTiter-Glo Luminescent Cell Viability Assay (Promega, G756A). Ramos-3F3 cells were cultured in a 96 well plate (0.1 mil/mL) and incubated with either vehicle control or 30 nM diCCP4-700DX for 90 min. The cells were then centrifuged (5 min, RT, 300 x g) and washed twice with complete medium. Subsequently, some conditions were exposed to 689 nm light (50 J/cm^2) and incubated overnight. The following day, the cell viability was measured after 1, 2 and 4h of addition of the XTT according to the manufacturer's protocol on a Tecan Spark M10 plate reader. In parallel, after 4h, the cell viability was measured using CTG on a Tecan Spark M10 plate reader.

Collagen plug experiments with Ramos-3F3. Collagen type I plugs with Ramos-3F3 cells were prepared with 20 µL MEM, 10 µL BIC, 150 µL collagen (PureCol collagen type I (Advanced BioMatrix)) and 90 µL Ramos-3F3 cell suspension (4.0 mil/mL) per well while on ice. This suspension was then seeded into a 48 well plate (Cellstar, 677180, lot# E220738T) and left in the incubator for 1h at 37 C and 5% CO_2. Subsequently, 750 µL of the appropriate concentration diCCP4-700DX in medium was added to each well and left for 1 to 16h in an incubator. The collagen plugs were then washed twice with complete medium and exposed to 689 nm light (50 J/cm^2, 290 mW/cm^2). After overnight incubation, the plugs were washed with PBS twice and each plug was detached with a blunt needle. The plugs were transferred to an Eppendorf tube and incubated with 500 µL of a mixture of DNAse I (1:100, Merck), collagenase D (1:100, Merck) and dispase II (1:100, Sigma) in PBS at 37°C on a roller bench to dissolve the plug. The cells were pelleted (RT, 300 x g, 5 min) and washed with medium. Finally, cell viability was measured using CTG.

Confocal microscopy of Ramos-3F3 collagen plugs. In separate collagen plugs, the cells were incubated with diCCP4-sCy5[1] for indicated times and subsequently washed with phenol red free complete medium twice. The collagen plugs were then detached with a blunt needle and transferred to an ibiTreat µ-Dish (Ibidi, 81156, lot#23010/1) containing 750 µL phenol red-free complete medium. Analysis was performed with the Leica SP8 AOBS microscope at 100x magnification, 37 C and 5% CO_2. All images are located in the center of the obtained z-stack.

FACS analysis of Ramos-3F3 collagen plugs with diCCP4-sCy5. Following the Ramos-3F3 collagen plugs protocol, the plugs were exposed to 10 nM diCCP4-sCy5[1] for 1, 2 and 16h and subsequently washed with phenol red free complete medium twice. The plugs were transported to an Eppendorf tube and 500 µL of a mixture of DNAse I (1:100) (Roche), collagenase IV (1:100) (Worthington) and dispase (1:100) (Roche) in PBS was added for 1h. The cells were pelleted (RT, 300 x g, 5 min) and washed with cold PBS twice. The cells were then fixed with cold 4% PFA (15min, RT), pelleted (5 min, 4 C, 600 x g) and washed with cold PBS twice. Samples were then analysed with the BD FACSverse and confocal microscope.

2. Supplemental figures

Figure S1: Chemical structure of diCCP4-700DX

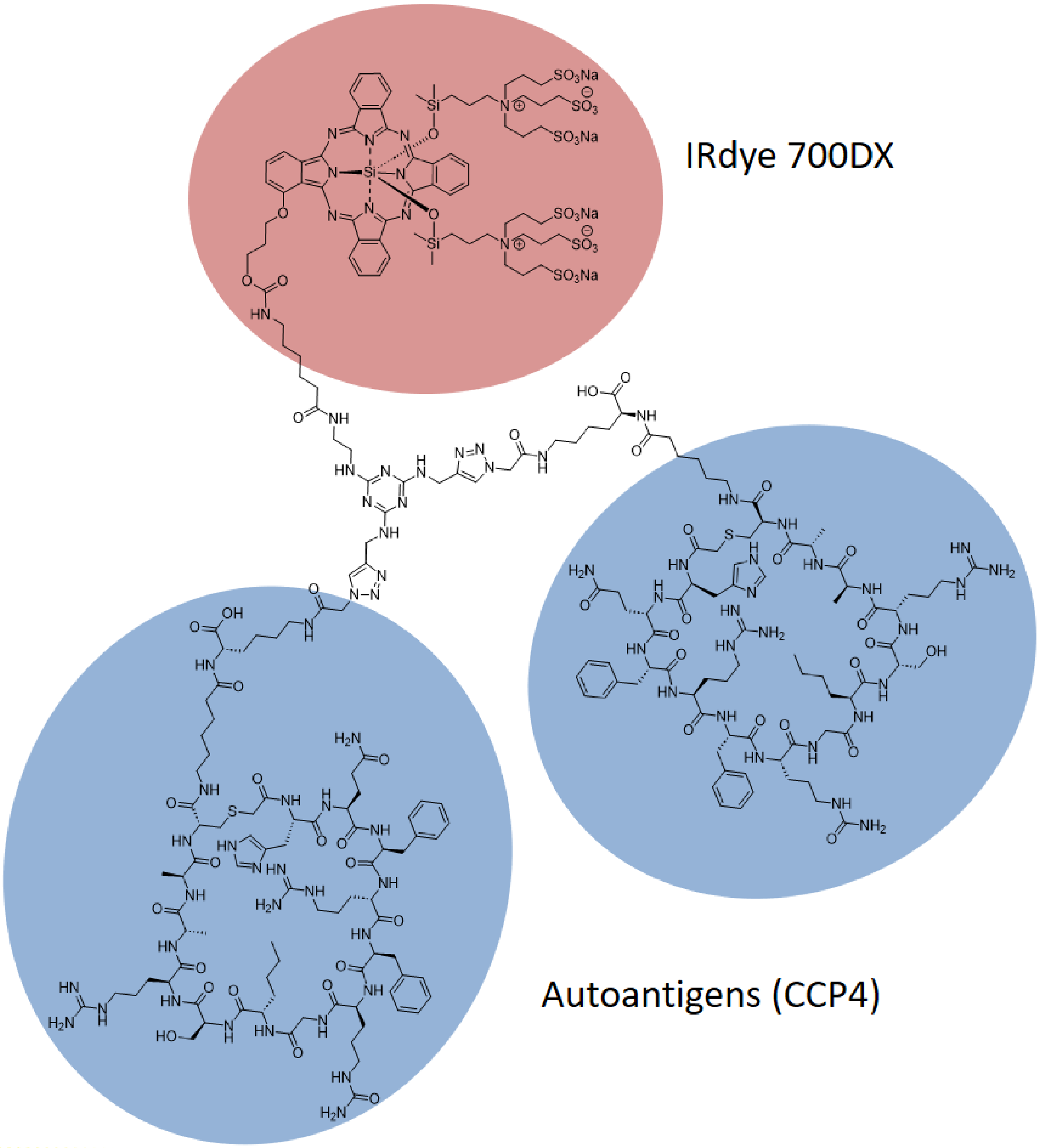

Figure S2: LC-MS analysis of diCCP4-700DX

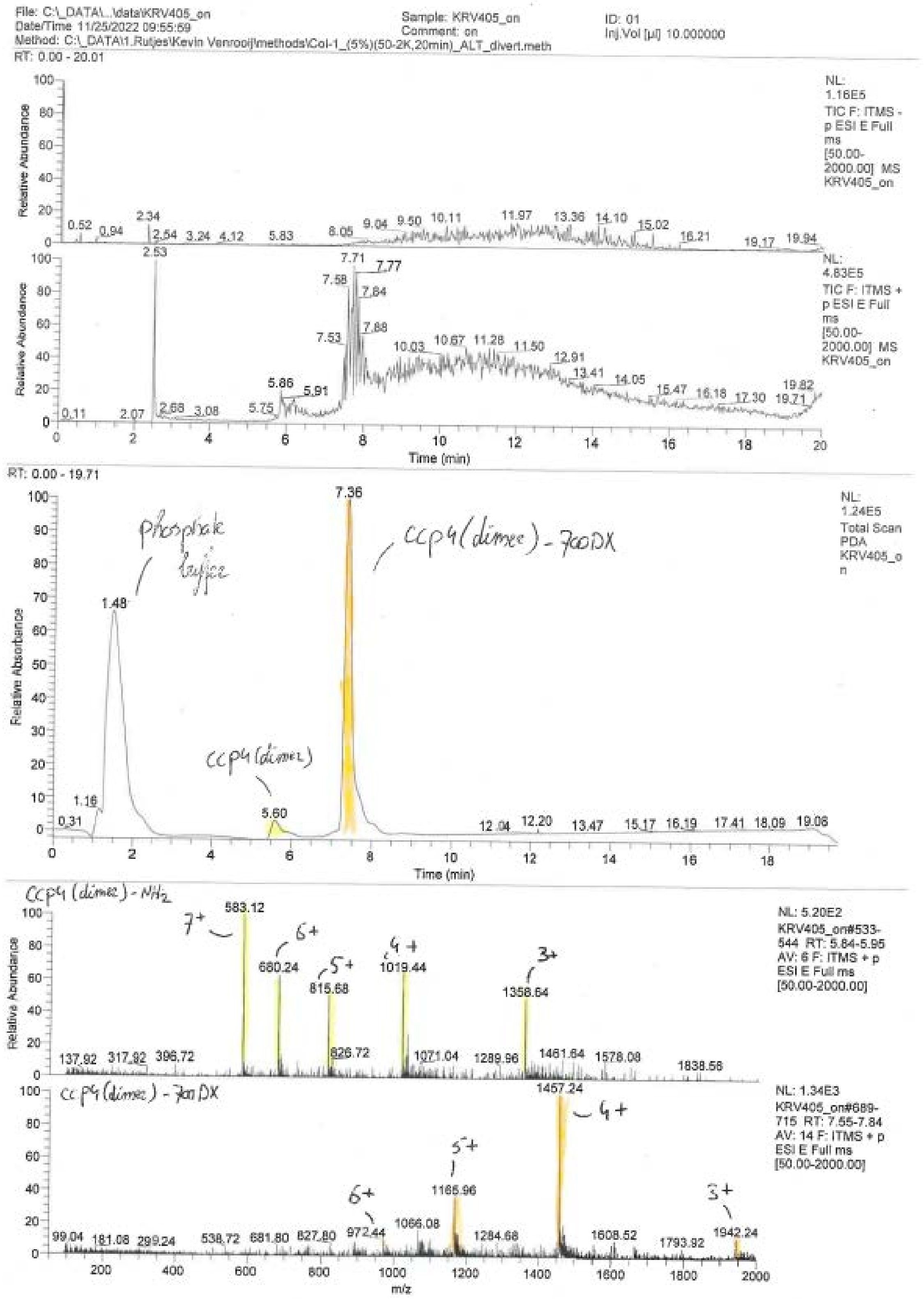

Fig. S2: *LC-MS analysis of KRV405. There is a trace amount of about 10% CCP4(dimer) left, as an excess was used. The acid labile silylether chains are still attached to the 700DX despite the acidic conditions.*

Figure S3: Light dosis at 689 nm does not affect Ramos-3F3 of TT cells

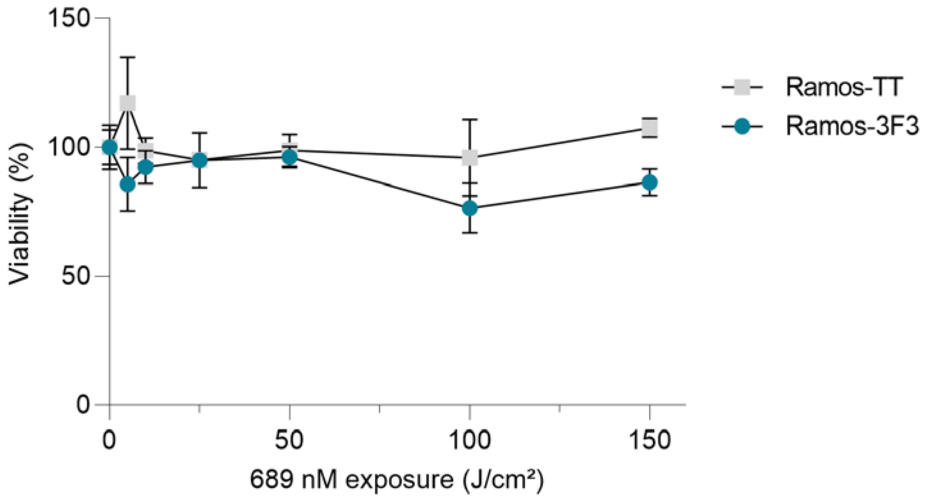

Fig. S3: *689 nm light (0-150 J/cm²) causes no adverse effect by the high dosage on Ramos-3F3 and Ramos-TT cells.*

Figure S4: Comparison of CTG and XTT methods for cell viability

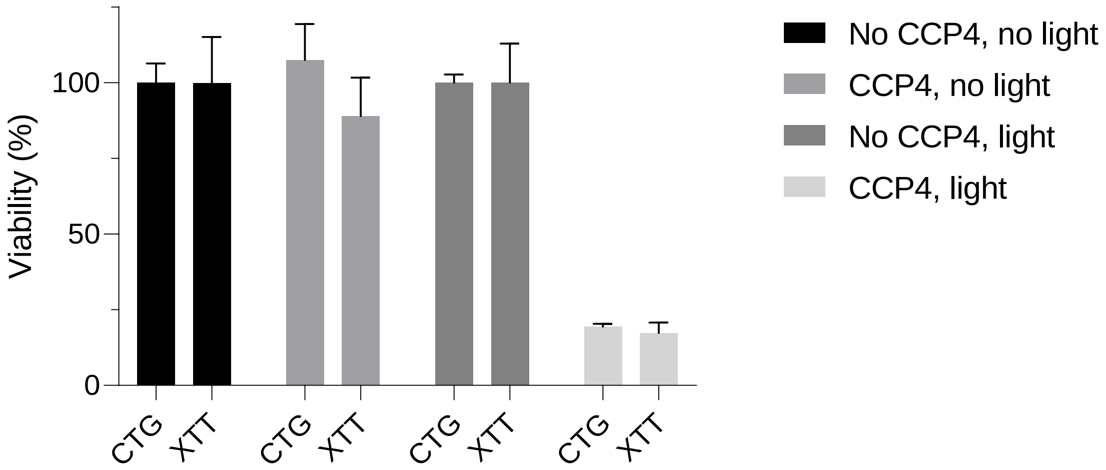

Fig. S4: *Comparison of CTG and XTT as read-out for cell viability after 90min of 10 nM diCCP4-700DX and 50 J/cm^2 689 nm. The CTG and XTT assays give a similar outcome, and the measured cell viability by CTG is thus not an artifact of the assay itself.*

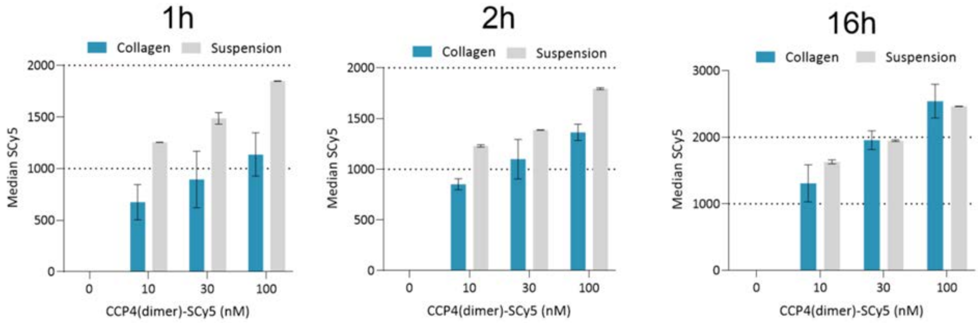

Figure S5: *MFI of diCCP4-SCy5 in collagen plugs and suspension cells over time at different peptide concentrations.*

Supplementary table S1: Antibodies used in flow cytometry experiments

Marker	Clone	Fluorochrome	Dilution	Supplier	function
**3F3 cells**		eGFP			
**CD3**	OKT3	PerCP-Cy5.5	1:100	Invitrogen	Pan T cells
**CD64**	10.1	PE	1:50	Biolegend	Monocyte activation
**BAFFR**	11C1	PE-Dazzle594	1:40	Biolegend	B cell activation
**CD25**	BC96	PE-Cy7	1:50	Biolegend	Late T cell activation
**CD56**	N901	APC	1:50	Beckman-Coulter	NK cells
**CD69**	FN50	AlexaFluor700	1:50	Biolegend	Early T cell activation
**CD19**	HIB19	BV605	1:25	Biolegend	B cells
**CD14**	M5E2	BV785	1:25	Biolegend	Classical Monocytes
**CD16**	3G8	BUV496	1:200	BD Biosciences	Non-classical Monocytes
**FVD**		ViaKrome808	1:1000	Beckman-Coulter	

Figure S6: FACS Gating strategy and panel overview

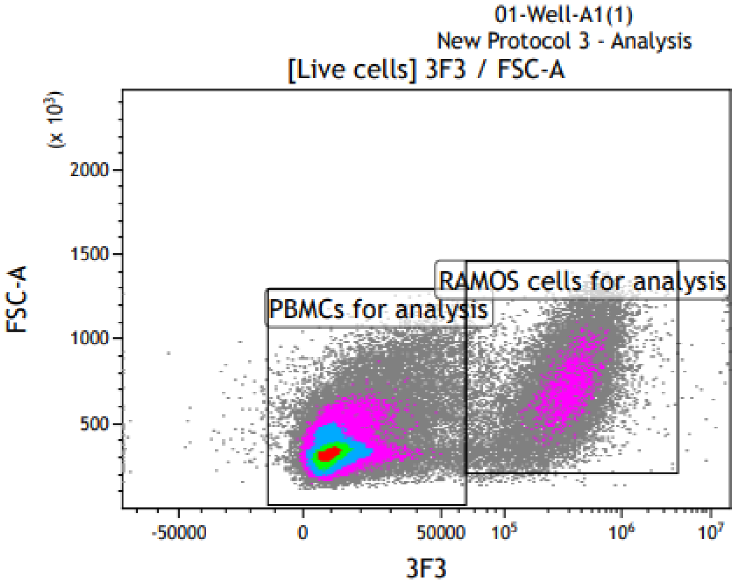

Gate	Number	%Total	%Gated	%GP Gated
All	109.913	73,28	100,00	N/A
PBMCs for analysis	90.699	60,47	82,52	72,83
RAMOS cells for analysis	18.994	12,66	17,28	15,25

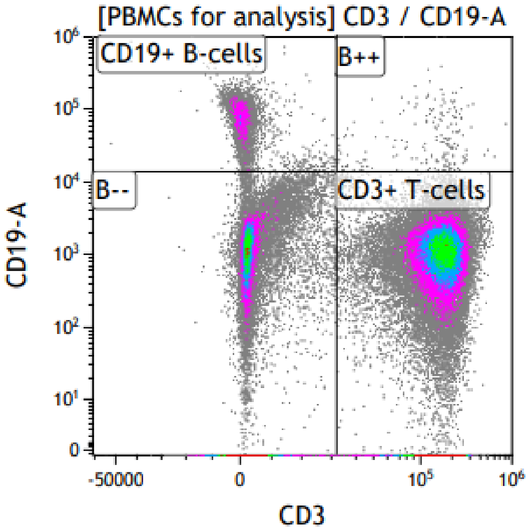

Gate	Number	%Total	%Gated
All	90.699	60,47	100,00
B--	29.155	19,44	32,14
B++	197	0,13	0,22
CD19+ B-cells	4.234	2,82	4,67
CD3+ T-cells	57.113	38,08	62,97

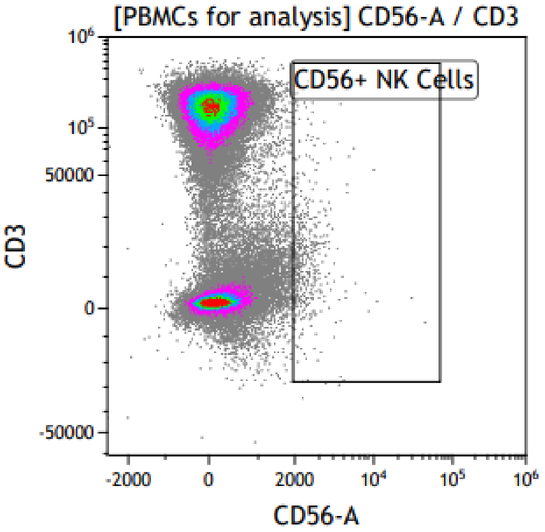

Gate	Number	%Total	%Gated
All	90.699	60,47	100,00
CD56+ NK Cells	489	0,33	0,54

01-Well-A1(1)
New Protocol 3 - Analysis

[CD3+ T-cells] CD69-A / CD25-A

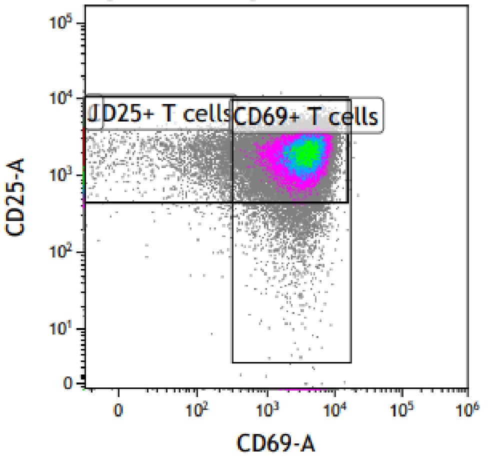

Gate	Number	%Total	%Gated
All	57.113	38,08	100,00
CD25+ T cells	54.377	36,25	95,21
CD69+ T cells	42.299	28,20	74,06
J	54.377	36,25	95,21

[PBMCs for analysis]
CD16-A / CD14-A

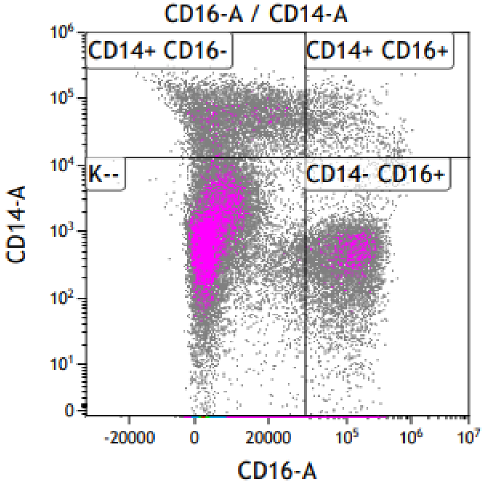

Gate	Number	%Total	%Gated
All	90.699	60,47	100,00
CD14- CD16+	9.153	6,10	10,09
CD14+ CD16-	5.364	3,58	5,91
CD14+ CD16+	882	0,59	0,97
K--	75.300	50,20	83,02

[CD19+ B-cells] BAFFR-A / CD64

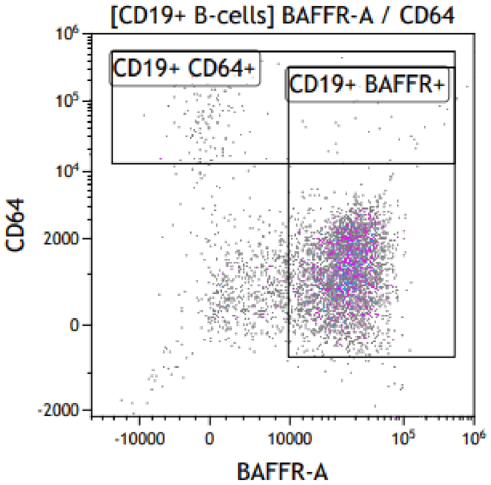

Gate	Number	%Total	%Gated
All	4.234	2,82	100,00
CD19+ BAFFR+	3.462	2,31	81,77
CD19+ CD64+	148	0,10	3,50

[CD14+ CD16-] CD14-A / CD64

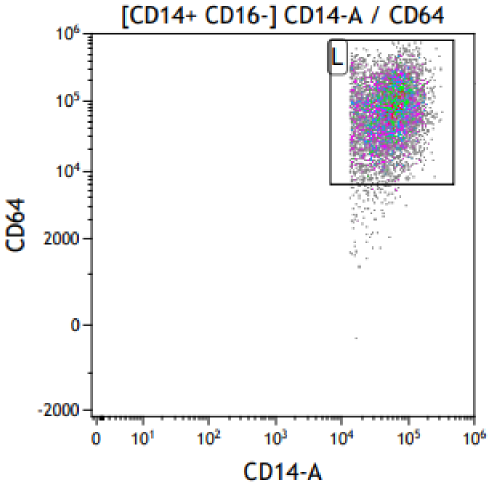

Gate	Number	%Total	%Gated
All	5.364	3,58	100,00
L	5.253	3,50	97,93

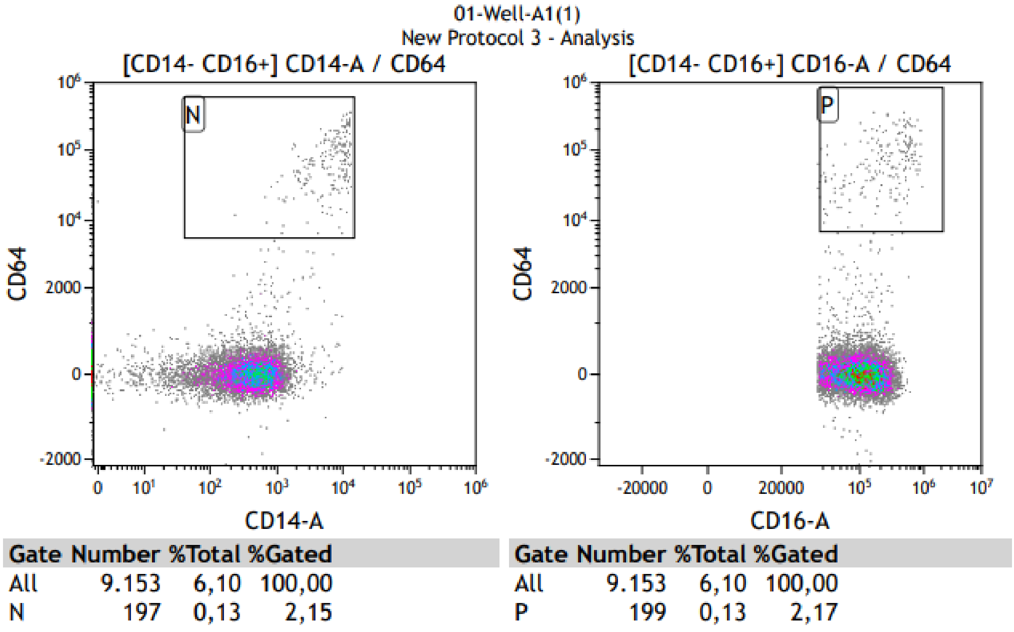

Figure S6: *Gating strategy to delineate the different (immune) cell populations in the collagen plugs and their activation marker expression.*

Figure S7: Expression pattern of CD56-APC in healthy PBMCs

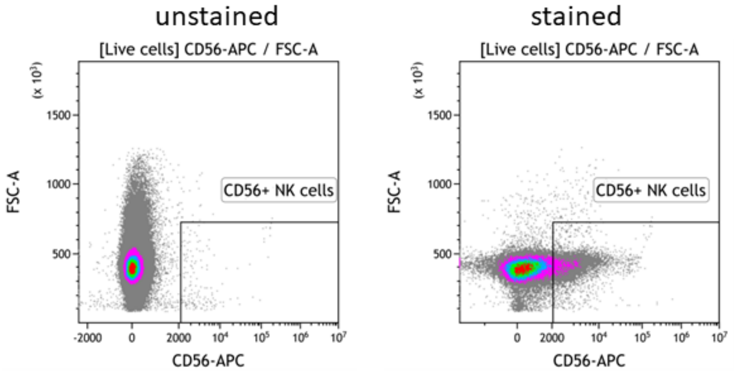

Figure S7: *Expression of CD56-APC in healthy-donor PBMCs was used to gate for CD56+ positivity in the gating strategy outlined in supplementary figure 7.*

Figure S8: Immune cell activation marker expression in Ramos-3F3 cells co-cultured with healthy donor PBMCs in collagen type I hydrogel.

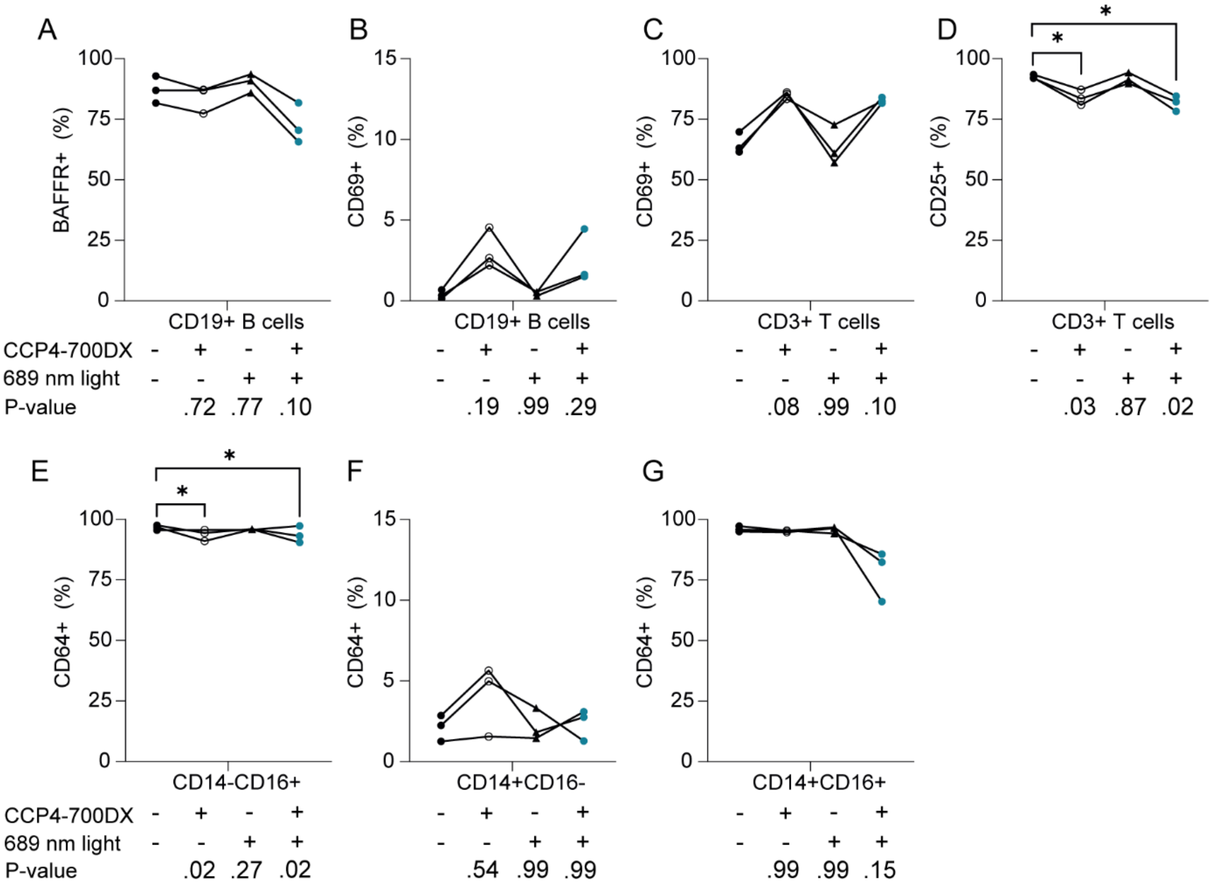

Figure S8: *Ramos-3F3 cells co-cultured with healthy donor PBMCs in collagen type I hydrogel. Conditions with diCCP4-700DX were incubated with 30 nM construct for 16h and light conditions received 50 J/cm² of 689 nm light. A) Percentage of BAFF-R+ cells in the CD19+ B cell population. B) Percentage CD69+ cells in the CD19+ B cell population. C) Percentage of CD69+ cells in the CD3+ T cell population. D) Percentage of CD25+ cells in the CD3+ T cells population. The percentage of CD64+ cells in CD14-CD16+ monocytes (E), CD14+CD16- (F) and CD14+CD16+ (G). * $p \leq 0.05$*

Figure S9: FACS analysis of healthy donor PBMCs treated with 689 nm light and diCCP4-700DX

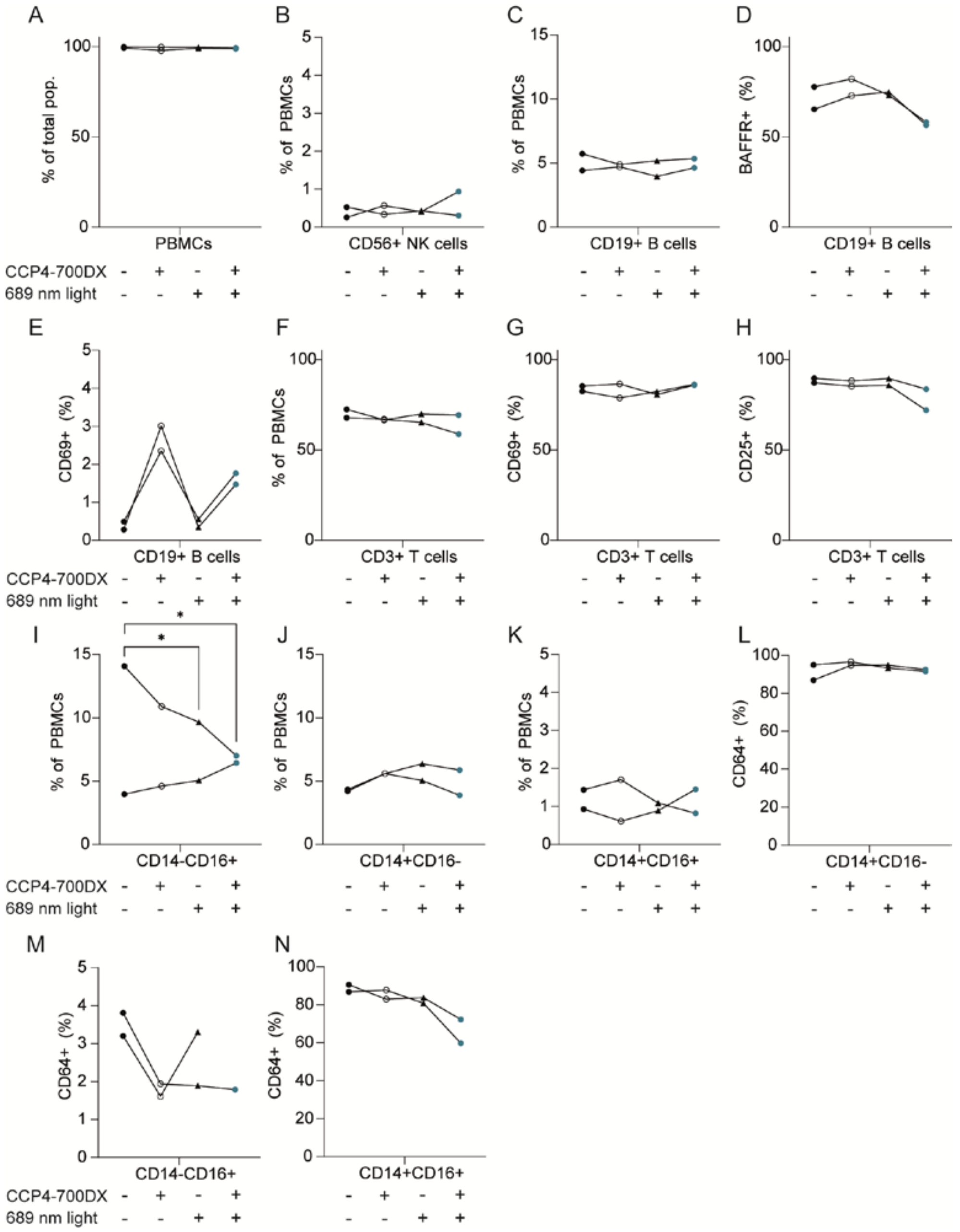

Figure. S9: *Healthy donor PBMCs in collagen type-I hydrogel. Conditions with diCCP4-700DX were incubated with 30 nM construct for 16 hours and light conditions received 50 J/cm*^{2} *of 689 nm light. A) The percentage of living PBMCs. Cells are not mistakenly identified as Ramos-3F3 with identical gating strategy as Figure 5. B) Percentage of CD56+ NK cells of the total PBMC population. C) Percentage of CD19+ B cells of the total PBMC population. D) Percentage of BAFFR+ cells in the CD19+ B cell population. E) Percentage CD69+ cells in the CD19+ B cell population. F) Percentage of CD3+ T cells of the total PBMC population. G) Percentage of CD69+ cells in the CD3+ T cell population. H) Percentage of CD25+ cells in the CD3+ T cells population. The percentage of monocyte subpopulations CD14-CD16+ (I), CD14+CD16- (J) and CD14+CD16+ (K) out of the total PBMC population. The percentage of CD64+ cells in CD14-CD16+ monocytes (L), CD14+CD16- (M) and CD14+CD16+ (N). * p ≤ 0.05.*
